# Trends in the incidence of dementia: design and methods in the Alzheimer Cohorts Consortium

**DOI:** 10.1007/s10654-017-0320-5

**Published:** 2017-10-23

**Authors:** Lori B. Chibnik, Frank J. Wolters, Kristoffer Bäckman, Alexa Beiser, Claudine Berr, Joshua C. Bis, Eric Boerwinkle, Daniel Bos, Carol Brayne, Jean-Francois Dartigues, Sirwan K. L. Darweesh, Stephanie Debette, Kendra L. Davis-Plourde, Carole Dufouil, Myriam Fornage, Leslie Grasset, Vilmundur Gudnason, Christoforos Hadjichrysanthou, Catherine Helmer, M. Arfan Ikram, M. Kamran Ikram, Silke Kern, Lewis H. Kuller, Lenore Launer, Oscar L. Lopez, Fiona Matthews, Osorio Meirelles, Thomas Mosley, Alison Ower, Bruce M. Psaty, Claudia L. Satizabal, Sudha Seshadri, Ingmar Skoog, Blossom C. M. Stephan, Christophe Tzourio, Reem Waziry, Mei Mei Wong, Anna Zettergren, Albert Hofman

**Affiliations:** 1 Department of Epidemiology, Harvard T.H. Chan School of Public Health, 677 Huntington Ave., Kresge 905, Boston, MA 02115 USA; 2 Channing Division of Network Medicine, Brigham and Women’s Hospital, Boston, MA USA; 3 Department of Epidemiology, Erasmus MC, Rotterdam, The Netherlands; 4 Department of Radiology and Nuclear Medicine, Erasmus MC, Rotterdam, The Netherlands; 5 Institute of Neuroscience and Physiology, Sahlgrenska Academy, University of Gothenburg, Gothenburg, Sweden; 6 Boston University School of Medicine, Boston, MA USA; 7 The Framingham Heart Study, Framingham, MA USA; 8 Department of Biostatistics, Boston University School of Public Health, Boston, MA USA; 9 INSERM, Univ Montpellier, Neuropsychiatry: Epidemiological and Clinical Research, Montpellier, France; 10 Cardiovascular Health Research Unit, Department of Medicine, University of Washington, Seattle, WA USA; 11 Kaiser Permanente Washington Health Research Institute, Seattle, WA USA; 12 University of Texas School of Public Health, Houston, TX USA; 13 University of Cambridge, Cambridge, UK; 14 Univ. Bordeaux, Inserm, Bordeaux Population Health Research Center, UMR 1219, Bordeaux, F-33000 France; 15 Department of Neurology, Memory Clinic, Bordeaux University Hospital, Bordeaux, France; 16 University of Texas Health Science Center at Houston McGovern Medical School, Houston, TX USA; 17 Icelandic Heart Association, Kópavogur, Iceland; 18 Faculty of Medicine, University of Iceland, Reykjavík, Iceland; 19 Department of Infectious Disease Epidemiology, School of Public Health, Imperial College London, London, UK; 20 Department of Epidemiology, Graduate School of Public Health, University of Pittsburgh, Pittsburgh, PA USA; 21 Laboratory of Epidemiology and Population Sciences, National Institute on Aging, Bethesda, MD USA; 22 Newcastle University, Newcastle upon Tyne, UK; 23 University of Mississippi Medical Center, Jackson, MS USA; 24 Departments of Neurology & Psychiatry, University of Pittsburgh, Pittsburgh, PA USA

**Keywords:** Alzheimer disease, Cohort analysis, Epidemiology, Consortium

## Abstract

Several studies have reported a decline in incidence of dementia which may have large implications for the projected burden of disease, and provide important guidance to preventive efforts. However, reports are conflicting or inconclusive with regard to the impact of gender and education with underlying causes of a presumed declining trend remaining largely unidentified. The Alzheimer Cohorts Consortium aggregates data from nine international population-based cohorts to determine changes in the incidence of dementia since 1990. We will employ Poisson regression models to calculate incidence rates in each cohort and Cox proportional hazard regression to compare 5-year cumulative hazards across study-specific epochs. Finally, we will meta-analyse changes per decade across cohorts, and repeat all analysis stratified by sex, education and APOE genotype. In all cohorts combined, there are data on almost 69,000 people at risk of dementia with the range of follow-up years between 2 and 27. The average age at baseline is similar across cohorts ranging between 72 and 77. Uniting a wide range of disease-specific and methodological expertise in research teams, the first analyses within the Alzheimer Cohorts Consortium are underway to tackle outstanding challenges in the assessment of time-trends in dementia occurrence.

## Introduction

It is estimated that approximately 47 million people are currently living with dementia, making it a leading cause of dependence and disability worldwide [1]. Because of a rapidly aging population, this number is predicted to have nearly doubled by 2040 [2]. Consequently the social and economic burdens of dementia are expected to substantially increase [3]. Yet, the projected burden of disease could be significantly lower if improvements in life conditions and health care over the last decades have had a beneficial effect on reducing risk of dementia. Indeed, recent studies in North America and Europe have reported a decline in the incidence of dementia over the last 20 years, up to 20% reduction per decade [4–8]. However, the underlying causes have not been determined, and discrepancies in described trends between sexes, and across different ethnicities and levels of education warrant further exploration [9, 10].

Valid assessment of time trends in the incidence of a disease calls for careful monitoring of it within the general population, in a consistent manner over a prolonged period of time. Population-based cohort studies are generally designed to establish determinants of disease, using consistent methodology throughout the course of data collection. The wide range of routinely collected data within these studies allows for exploration of effect modifiers (e.g. genotype or sex), as well as various potential underlying causes, such as changes in cardiovascular risk management, comorbidity (e.g. stroke), and level of education. Worldwide, however, only a limited number of studies exist, that are carried out in unselected populations and provide the infrastructure and decade-long follow-up duration necessary to determine trends in dementia incidence. Power and precision of these individual studies are not always sufficient to answer the research questions outlined above. We therefore aim to jointly analyse available long-term population-based data seeking confirmation for any time trends in dementia occurrence and importantly identify determinants of such trends. The results will have important implications for informing public health policy focused on dementia reduction.

## Materials and methods

### The Alzheimer Cohorts Consortium

The Alzheimer Cohorts Consortium is a collaboration of nine prospective cohorts studies from the United States and Europe including: the Age, Gene/Environment Susceptibility (AGES)-Reykjavik Study, the Atherosclerosis Risk in Communities (ARIC) study, the Cardiovascular Health Study (CHS), the Cognitive Function and Ageing Studies (CFAS), the Framingham Heart Study (FHS), the Gothenburg population studies, the Personnes Agées QUID (PAQUID) study, the Rotterdam Study, and the Three-City Study (3C). All cohorts are population-based and comprise of prospectively collected data on dementia (and in most studies information on clinical subtypes), in addition to genotyping, and extensive (cardiovascular) phenotyping.

### Description of cohorts

A summary of the key characteristics of each cohort are presented in Table 1. Across the cohorts there are more than 70,000 individuals of whom around 6300 have developed dementia to date. Briefly, the *AGES*-*Reykjavik Study* represents a sample drawn from the population-based Reykjavik Study [11]. The original Reykjavik Study comprised a random sample of 30,795 men and women born between 1907 and 1935 and living in Reykjavik in 1967. Between 1967 and 1996, six examinations were conducted in six sub-cohorts, and 5764 survivors of the original cohort were re-examined for the AGES-Reykjavik study between 2002 and 2006. The *ARIC study* is a population-based prospective cohort study of cardiovascular disease and its risk factors [12]. Chosen by probability sampling from four U.S. communities including Winston-Salem (NC), Jackson (MS), Minneapolis (MN), and Baltimore (MD), the study included 15,792 individuals aged 45–64 years at study baseline in 1987–1989. Participants completed four clinic examinations, conducted 3 years apart, up till 1998, and undergo annual follow-up for clinical events. Between 2011 and 2013, all surviving ARIC participants were invited to a 5th visit (ARIC Neurocognitive Study (ARIC-NCS), when a comprehensive dementia assessment was performed. The *CHS* is a population-based cohort study of risk factors for cardiovascular disease in adults aged 65 years and older, recruited in 1989–1990 from random samples of the Medicare lists in four U.S. field centers, namely Sacramento (CA), Hagerstown (MD), Winston-Salem (NC), and Pittsburgh (PA) [13] The original predominantly white cohort of 5888 persons was expanded by enrolment of 687 African-Americans in 1992–1993. Participants completed standardized clinical examinations and questionnaires at study baseline and at annual follow-up visits until 1999. Ongoing follow-up for clinical events occurs by phone every 6 months thereafter. The *CFAS* comprise two separate population-based studies in three sites from the original Medical Resource Council CFAS (Cambridgeshire, Newcastle and Nottingham), conducted 20 years apart in the UK [5]. The samples includes individuals aged 65 years and over regardless of residential status (i.e. persons living in the community as well as institutions). The first study, CFAS I, recruited in 1990-1993 (N = 7635) with a 2-year follow-up in of 20% of participants (n = 900) in 1993–1995. A second, comparison study, CFAS II, was initiated two decades later, between 2008 and 2011 (N = 7796) with a 2-year follow-up screening (n = 5288) in 2011–2013. Identical methods were used in CFAS I and CFAS II with the exception of a two-stage enrolment (separate screening and assessment interview) utilized in CFAS I and a one-stage enrolment for CFAS II (single screening and assessment interview) [14]. The *FHS* began in 1948 with the recruitment of an original cohort of 5209 men and women who were aged 28–62 at study entry [15]. In 1971, a second generation of study participants, including 5124 children and spouses of children of the original cohort were enrolled [16]. Enrolment of the third generation cohort of 4095 children of offspring cohort participants began in 2002 [17]. Clinic follow-up examinations take place approximately every two year for the original cohort and approximately every 4 years for the Offspring and Third Generation cohorts. In addition, the cohorts are under continuous surveillance for disease endpoints, such as myocardial infarction, stroke, and dementia. The *Gothenburg population studies* consist of data from four studies which recruited individuals representative of the Swedish population [18]. These include Kvinnoundersökningen (KVUS), a study of 1462 women aged 38–60 who have been followed since 1968; the H70 study, which includes representative samples of 70-year-olds born 1906–1907 (N = 414), recruited 1976–1977, and followed until death, and 1930 (N = 522), recruited 2000–2001 and followed until now, the H85 study, which includes samples of 85-year-olds born 1901–1902 (N = 494), first examined in 1986 and followed until death, and 1923–1924 (N = 571), first examined in 2008–2009 and followed until age 90; and the 95-plus study that started in 1996 and by 2012 had recruited a total of 950 individuals. The *PAQUID* cohort is a population-based study in the southwest of France of 3777 individuals aged 65 years or older recruited in 1988 [19]. There have been nine waves of data collection at 1, 3, 5, 8, 10, 13, 15, 17, 20, 23, 25, and 27 years after the baseline assessment. The *RS* is a prospective population-based cohort study comprising 14,926 subjects aged 45 years or older [20]. Baseline data of 7983 participants were collected between 1990 and 1993, with subsequent cohort expansions in 2000 (3011 individuals) and 2006 (3236 individuals). Participants have been examined once every 4 years. In addition, the entire cohort is continuously under surveillance for disease outcomes through linkage of electronic medical records with the study database. The *3C* is a longitudinal population-based study of the relation between vascular diseases and dementia in persons aged 65 years and older [21]. Between 1999 and 2001, a total of 9294 non-institutionalized persons were recruited from the electoral rolls of three French cities: Bordeaux (South-West), Dijon (North-East) and Montpellier (South-East). Participants have been re-examined every 2 years.

**Table 1:** Description of the cohorts included in the Alzheimer Cohorts Consortium

Study	AGES-Reykjavik	ARIC NCS	CFAS I/II	CHS	Framingham Heart Study	Gothenburg studies	PAQUID	Rotterdam Study	Three-City Study
Country	Iceland	USA	UK	USA	USA	Sweden	France	Netherlands	France
Study baseline	2002	2011–2013	1991/2008	1991	1990	1990	1988	1990	1999
Family-based	No	No	No	No	Yes	No	No	No	No
Study sites	1	4	3/3	4	1	1	1	1	3
Dementia follow-up (years)	14	5^a^	2/2	18	25	25	27	25	16
Diagnosis of dementia	DSM-IV	DSM-V	DSM-IIIR	DSM-IV	DSM-IV	DSM-IIIR	DSM-IIIR	DSM-IIIR	DSM-IV
Diagnosis of AD	NINCDS-ADRDA	NIA-AA	N/A	NINCDS-ADRDA	NINCDS-ADRDA	NINCDS-ADRDA	NINCDS-ADRDA	NINCDS-ADRDA	NINCDS-ADRDA

*AGES* Age, Gene/Environment Susceptibility, *ARIC-NCS* Atherosclerosis Risk in Communities Neurocognitive Study, *CFAS* Cognitive Function and Ageing Studies, *CHS* Cardiovascular Health Study, *PAQUID* Personnes Agées QUID, *AD* Alzheimer’s disease, *N/A* not available

^a^Efforts to work-up recent incident dementia cases are ongoing as of January 2017

### Ethics

All participating studies have ethical approval, and all subjects (or their nominated representative) provided written informed consent.

### Dementia assessment

The primary outcome is all-cause dementia and this is assessed in all cohorts (Table 1). The secondary outcome is diagnoses of Alzheimer’s disease (AD), the most common clinical subtype. Methods for dementia diagnosis varied between cohorts, but are consistently applied in each cohort throughout the study period. An exception is CHS, in which participants are re-examined more frequently from 2002 onwards (i.e. annually) compared to before diagnosis of dementia and is based on change in cognition and function from previous visits.

### Defining epochs

One option for assessing trend over time is to define units of time based on the same calendar years across cohorts. This method makes it easy to combine results across cohorts, but ignores the fact that each study has its own pattern of examination cycles and therefore risks bringing in more biases based on study design. To avoid this, we choose to define units of time, or epochs, specific to each study based on each interview wave. This allows us to take full advantage of all available data in each study, maximize the person-years available and also, by using the median time since beginning of first epoch (as described in more detail below in the statistical analysis section), we can compare trends over the years across all the cohorts. Requirements for defining an epoch are: (1) start at or close to an examination cycle, (2) non-overlapping with previous or subsequent epoch, and (3) at least 5 years in length. Participants need to be 60 years or over, and free of dementia at the start of the epoch to be included. All cohorts have follow-up for at least two epochs, except for AGES, in which only a baseline epoch has sufficient follow-up.

### Statistical analysis

All analyses are currently being performed in individual cohorts and results will be meta-analysed when appropriate. Demographic characteristics of each cohort are summarized using means with standard deviation (SD) for continuous measures and frequencies for categorical measures. The calendar time-window of the present analyses is restricted to 1990–2015 to allow for assessment of incidence rates and time trends across the same time-period in all cohorts.

Five-year incidence rates (IRs) with 95% confidence intervals (CI) are being calculated using age-adjusted Poisson regression models. Groups are first stratified by 5-year age-groups and then additionally by sex. IRs are reported for the middle age within each age group, e.g. 62.5 for the (60–65) age group, 67.5 for the (65–70) age group, etcetera. A participant is included in a particular age group if they were dementia-free at start of the age group category. Since all the cohorts have repeated visits with participants, when data was available, a single person could contribute to IRs of multiple age groups. To account for this, we employ robust sandwich estimators to calculate the 95% CI around the IRs.

Five-year cumulative hazards and hazard ratios are being assessed individually in each cohort and not combined across studies because of differing timing of examinations. Non-overlapping epochs are defined based on examination cycles and are specific to each cohort. Five-year cumulative hazard and hazard ratios (HRs) are being calculated using a Cox proportional hazard regression model and adjusted for age and sex in non-stratified models using a robust sandwich estimator for covariance structure [22]. Participants who did not experience a dementia diagnosis are censored at the last date they were known to be free of dementia, or 5 years after the beginning of the epoch, whichever was sooner. Hazard ratios are being computed for each epoch as compared to the first epoch followed by trend per decade. We do this by assigning to each epoch an index value equal to median time in years since the beginning of the first epoch. For example, if epoch 1 was 1995–1999 and epoch 2 was 2000–2005 then the index variable would be 2.5 and 7.5 respectively. The index variable is then used in the Cox proportional hazard regression to assess a linear change in hazard of dementia over the epochs or linear trend. To ensure the analyses are identical across cohorts, statistical code using both SPSS and SAS was developed and tested using the Rotterdam Study dataset to ensure results matched between statistical software programs and then provided to each cohort for analyses. All analyses are currently being performed using either SPSS version 23.0 (IBM Corp, Armonk, NY, USA) or SAS 9.4 (Cary, NC, USA).

## Results

Descriptions of all cohorts are summarized in Table 1. In all cohorts combined there is data on almost 69,000 people at risk of dementia with the range of follow-up years between 2 and 27. The average age at baseline is similar across cohorts ranging between 72 and 77 (Table 2). Each cohort is made up of > 50% females, ranging from 56.8% in FHS to 76.3% in the Gothenburg studies (Table 2). All cohorts collect information on incident dementia and all but one cohort (CFAS I/II) also collecting information on incident AD.

**Table 2:** Demographics of the cohorts included in the Alzheimer Cohorts Consortium

Study	AGES-Reykjavik	ARIC NCS	CFAS I/II	CHS	Framingham Heart Study	Gothenburg studies	PAQUID	Rotterdam Study	Three-City Study
At risk of dementia	5722	6538	7635/7762	2798	8586	3024	2997	11,044	8250
Mean age (years)	77.0	75.8	75.0/76.4	74.7	72.1	77.3	75.3	72.0	74.0
Women (%)	57.7	58.8	61.6/56.1	59.1	56.8	76.3	58.0	58.5	61.3
Caucasian ethnicity (%)	100	76.1	99.1/97.2	89.5	100	100	NC^b^	98.0	100
Incident dementia^a^	250	344	250/250	680	800	700	940	1400	950
Incident AD^a^	150	72	N/A	590	510	300	730	1100	650

*AGES* Age, Gene/Environment Susceptibility, *ARIC-NCS* Atherosclerosis Risk in Communities Neurocognitive Study, *CFAS* Cognitive Function and Ageing Studies, *CHS* Cardiovascular Health Study, *PAQUID* Personnes Agées QUID, *AD* Alzheimer’s disease, *N/A* Not Available

^a^Approximation of total number of individuals with dementia per cohort at time of press

^b^Not collected

## Discussion

Several of the cohorts within the Alzheimer Cohorts Consortium have previously published data on time trends in the prevalence and incidence of dementia [4, 5, 7, 8]. In this collaboration, we aim to reproduce these findings using consistent analytical techniques, and harmonise results across the individual cohort studies to identify underlying trends and investigate subgroups of interest (e.g. stratification by gender) and effect modifiers. The close collaboration between cohorts in the consortium, along with the high-quality study design and data collection methods facilitate these analyses of incidence trends over the past three decades.

### Cohort enrolment, resampling, and survival bias

Most cohorts contributing data to these analyses use a closed-cohort design with single enrolment, while two of the cohorts, FHS and the Rotterdam Study, are expanded during the study period, including additional individuals from the source population and one set of cohorts, CFAS I and CFAS II is composed of two comparison cohorts each with a follow-up assessment. Single enrolment in closed cohorts will limit the number of comparable individuals within the same age range, as the cohort on average becomes older over time. We intend to utilize the full potential of this collaboration by including all available data, i.e. expansion cohorts as well as the originally defined cohorts. On a participant level, we allow a single participant to be included in multiple epochs as long as they are free of dementia at the start of the epoch. This can lead to underestimation of the standard error and thus we utilize robust standard error estimates. Restricting non-demented participants to only a single epoch, such as the epoch of their first examination, would deplete the number of participants susceptible to dementia over time. This would mean that individuals at high risk would be underrepresented at later time points. Such selection bias could result in underestimation of the incidence rates and cumulative hazards in more recent years. Conversely, mortality rates have dropped substantially over the past decades, and the increase in life-expectancy renders more people susceptible to dementia these days than in earlier years. This survival bias may cause underestimation of a declining trend in the incidence of dementia.

### All-cause dementia as a primary outcome measure

Distinguishing clinical AD from other dementia subtypes such as vascular dementia or dementia with Lewy bodies has proven challenging in light of the multiple pathologies co-occurring with increased age in the majority of cases with dementia [23–26]. This is particularly troubling as the incidence of dementia increases steeply with age, with the vast majority of dementia cases occurring after 70 years of age. Studies of dementia and sporadic AD focused on older aged samples consequently recruit individuals in whom a large number of factors (e.g. neurodegenerative and vascular) contribute to cognitive decline and dementia, hampering accurate diagnosis of dementia subtypes. Not only does this burden etiological research, it could also contribute to heterogeneity in dementia diagnoses between cohorts. In addition, diagnosis of all-cause dementia is less susceptible to changes in clinical subtyping of dementia that may have occurred over time. For these reasons, the focus of the analysis is on all-cause dementia, which can be more reliably defined across cohorts. The wide age range of the unselected populations guarantees generalizability to understudied elderly individuals, and reflects the full spectrum of the dementia burden in the population.

### Dementia occurrence across cohorts

Despite many similarities in design and data collection between the cohorts in this collaboration, there are also factors that may lead to differences in baseline incidence rates across the different cohorts. These include underlying population traits (e.g. access to health care, socioeconomic status, genetic make-up, and lifestyle), and variations in methodology (e.g. re-examination interval, continuous surveillance methods). For the most part these are likely to remain constant over the course of the study period, and although contributing to differences in baseline incidence, arguably less likely to influence within study trends. Differences in risk of mortality across cohorts, however, may differentially affect the results, because of survival bias, as described above. In addition, differences in the diagnosis of dementia across cohorts and region-specific changes in the clinical assessment of dementia over time pose a challenge to trend analysis. Lastly, all cohorts are embedded within the general population, but cannot completely avoid variation in sampling strategies and inclusion rates. Moreover, strategies for follow-up and disease surveillance vary, potentially affecting attrition or diagnostic sensitivity, which may hamper absolute risk estimates in particular. Variation may, in part, be addressed by accounting for genetic heterogeneity, further stratification when sample size allows (e.g. for educational attainment, vascular disease burden), or use of more advanced statistical methods, such as illness-death modelling to deal with death occurring during the inter-examination interval.

Within the Alzheimer Cohorts Consortium nine prospective population-based cohort studies leverage conscientiously collected data over a 25-year period with the aim to determine trends in the incidence of dementia and to unravel underlying causes. Uniting a wide range of disease-specific and methodological expertise in research teams within and beyond these cohorts, the first analyses within the Alzheimer Cohorts Consortium are underway to tackle outstanding challenges in the assessment of time-trends in dementia occurrence.
